# Mesenchymal stem cell therapy for ischemic stroke: Novel insight into the crosstalk with immune cells

**DOI:** 10.3389/fneur.2022.1048113

**Published:** 2022-11-08

**Authors:** Nana Tan, Wenqiang Xin, Min Huang, Yuling Mao

**Affiliations:** ^1^Department of Health Management, The Third Affiliated Hospital of Guangzhou Medical University, Guangzhou, China; ^2^Department of Neurosurgery, Tianjin Medical University General Hospital, Tianjin, China; ^3^Department of Obstetrics and Gynecology, Center for Reproductive Medicine, Guangdong Provincial Key Laboratory of Major Obstetric Diseases, The Third Affiliated Hospital of Guangzhou Medical University, Guangzhou, China; ^4^Key Laboratory for Reproductive Medicine of Guangdong Province, The Third Affiliated Hospital of Guangzhou Medical University, Guangzhou, China

**Keywords:** ischemic stroke, mesenchymal stem cells, immunomodulation, preclinical study, clinical trials

## Abstract

Stroke, a cerebrovascular accident, is prevalent and the second highest cause of death globally across patient populations; it is as a significant cause of morbidity and mortality. Mesenchymal stem cell (MSC) transplantation is emerging as a promising treatment for alleviating neurological deficits, as indicated by a great number of animal and clinical studies. The potential of regulating the immune system is currently being explored as a therapeutic target after ischemic stroke. This study will discuss recent evidence that MSCs can harness the immune system by interacting with immune cells to boost neurologic recovery effectively. Moreover, a notion will be given to MSCs participating in multiple pathological processes, such as increasing cell survival angiogenesis and suppressing cell apoptosis and autophagy in several phases of ischemic stroke, consequently promoting neurological function recovery. We will conclude the review by highlighting the clinical opportunities for MSCs by reviewing the safety, feasibility, and efficacy of MSCs therapy.

## Introduction

Stroke is responsible for almost six million deaths, at least 10% of all mortalities yearly, and two-thirds of stroke survivors remain disabled (1). Worldwide, over 80 million people have survived a stroke; 70% of incident strokes are ischemic (1). Although recent evolutions of thrombectomy technology, as well as improvements in imaging devices, have achieved ground-breaking changes in ischemic stroke therapy (2), given its narrow therapeutic time window and the concern of hemorrhagic complications (3), thrombolysis is still not performed routinely (4). In this context, it is urgent to yield neurorestorative treatments for abrogating stroke-induced neurological deficits for both basic scientists and clinical researchers. Cell therapy is emerging as a promising novel modality for facilitating neurologic recovery after a stroke (5). Harnessing the immune system to function and effectively boost neurologic recovery has transitioned from a theoretical possibility to a viable therapeutic option for ischemic stroke. Mesenchymal stem cells (MSCs) transplantation is an attractive therapy method because they have the potential for proliferation, differentiation, and immunomodulatory properties (6, 7). While the MSCs can be derived from any type of tissue beyond the bone marrow, adipose, and placenta, these MSCs share the same core attributes of ability to cell migration patterns and behave as immunomodulatory cells (8–10). In addition to immunomodulation, growing evidence demonstrates that MSCs are involved in multiple pathological processes by targeting series downstream. Such downstream activities include the inhibition of apoptosis and autophagy and the promotion of angiogenesis, neurogenesis, and synaptic remodeling in several phases of ischemic stroke (11, 12). MSCs may also be an ideal candidate for cell transplantation therapy for ischemic stroke. Despite growing evidence indicating that MSCs may improve neurological function under pathological conditions, including stroke (13, 14), data on the interaction between MSCs and immunomodulation is limited. In this review, we summarize the therapeutic effects of MSCs both in preclinical studies and in clinical stroke trials. We also consider the mutual crosstalk between MSCs and immune cells under stroke conditions.

## Mesenchymal stem cells

Rodent bone marrow cells were first ectopically transplanted into the kidney capsule by Friedenstein et al. in the 1960s and 1970s, showing an osteogenic effect (15). Given the potential to differentiate into various cell lineages, Caplan et al. suggested the “mesenchymal stem cells” term in 1991 (16, 17). MSCs are multipotent fibroblast-like cells that, interestingly, exist in various adult tissues, including adipose tissue, periosteum, liver, spleen, muscle connective tissue, placenta, umbilical cord blood, dental pulp, and aborted fetal tissues (18–20). Further, The Mesenchymal and Tissue Stem Cell Committee of the International Society for Cellular Therapy (ISCT) recommended specific minimum MSC criteria to distinguish them from other cell types by expression of many cell surface markers, including CD73, CD90, and CD105, and the absence of expression of CD45, CD34, CD14, CD19, CD11b, or Human Leukocyte Antigen–DR isotype (21–23). Recently, a significant number of novel cell surface markers associated with the stemness within MSCs, namely SSEA1/4, CD44, CD146, and CD271, have been revealed as well (23–26). A further two criteria are that isolated cells show adherence to plastic in culture and the capacity to differentiate into adipocytes, osteoblasts, and chondroblasts *in vitro* (21–23). To date, MSCs have become the most widely studied stem cell population and are studied in various preclinical models and clinical settings alike. And these studies have focused on the vital roles in coordinating tissue responses to ischemic stroke in acute and post-acute stroke settings, in which MSCs modulate cell survival, cell apoptosis, autophagy angiogenesis, and immunosuppression (23), consequently supporting neurological recovery.

## Therapeutic application of MSCs in preclinical ischemic stroke study

### MSCs promote post-stroke cell survival

Upon an ischemic stroke, the cerebral artery occlusion influences the survival of various brain cells, such as brain neurons, glial cells, and vascular cells. Among these cells, the neurons are the most vulnerable, and neuronal viability plays a crucial role in neurological recovery after ischemic stroke (27, 28). Studies in experimental models mimicking ischemic stroke imply that MSCs can abrogate ischemia-induced neuronal survival and neurological function recovery. As such, under such conditions, MSCs derived from bone marrow, adipose tissue, and umbilical cord can significantly reduce neuronal death (29–31). In addition, neurological recovery is also associated with the successful restitution of vascular and glial functions. During the ischemic lesion remodeling, neurons, glial cells, and vascular cells can strongly interact with each other, contributing to neurological recovery (27). Interestingly, it is demonstrated that MSCs are involved in promoting the survival of microglia, astrocyte, and endotheliocyte survival via regulating many pathways (32–35). Notably, white matter demyelination predates axonal injury in the early stage of ischemic stroke, indicating a time window for stroke intervention focusing on preventing or postponing axonal injury through myelin regeneration (36). Meanwhile, Bagdasarian et al. (37) applied therapeutic MSC to a rodent stroke model and demonstrated their efficacy in white matter by comparison of Diffusion tensor imaging and Neurite Orientation Dispersion and Density Imaging metrics. MSCs exert many unique biological effects, including self-recovery via promoting post-stroke cell survival, providing a promising cellular therapeutic approach for treating white matter injury (38).

### MSCs suppress post-stroke cell apoptosis

Among the many cell death pathways (39), apoptosis accounts for a large proportion of cell death under such a condition (40), a rational and reactive performance made to sacrifice specific cells for the benefit of the tissue. Researchers have indicated that MSCs have vital roles in regulating cell apoptosis. For example, Kong et al. (41) demonstrated MSCs potentially protect the cortical neurons from OGD injury *in vitro* by rescuing neurons from apoptosis. Xiao et al. (42) indicated that bone marrow-derived MSC-exosomes repressed oligodendrocyte apoptosis via releasing exosomal miR-134, in turn negatively regulating the caspase-8-dependent apoptosis pathway. In addition to apoptosis, MSCs can promote cell survival by alleviating parthanatos and necroptosis. By co-culturing MSCs with hypoxic neurons, Kong et al. indicated that MSCs prevented neurons from parthanatos by suppressing the expression of nuclear translocation of apoptosis-inducing factors (41). The reduction of neuronal necrosis kinase RIP1 and RIP3 levels caused by MSCs, meanwhile, was tightly related to the suppression of neuronal necroptosis (41).

### MSCs suppress post-stroke cell autophagy

Autophagy, another type of cell death, is an evolutionarily conserved cellular mechanism that balances cellular nerve homeostasis. It is a process that results from the injury in cells' internal conditions, including starvation, hypoxia, and infection (43). MSCs can inhibit autophagy and, in turn, promote cell survival. Kuang et al. (31) illustrated that the application of adipose-derived MSC-exosomes suppressed the autophagic response under both *in vitro* hypoxia and *in vivo* cerebral ischemia regarding cell survival through transferring of miR-25, as a consequence, supporting post-stroke neurological function recovery. Moreover, the knockdown of SNHG12 in MSCs boosted the effects of MSCs in suppressing hypoxia-induced autophagy in brain microvascular endothelial cells and MCAO rats by interacting with the PI3K/AKT/mTOR signaling pathway (44). By contrast, MSCs can reverse ischemic injury by enhancing autophagy as well (45, 46). Likewise, Zeng et al. indicated that PC12 cells were exposed to oxygen-glucose deprivation (OGD) and cocultured with MSCs secreted extracellular vesicles (EVs). Under such conditions, MSC-secreted EVs significantly attenuated pyroptosis mediated by NLRP3 inflammasome by promoting AMPK-dependent autophagy flux (47).

### MSCs promote post-stroke angiogenesis

During post-stroke conditions, capillaries are dysfunctional, and blood-brain barrier permeability is increased, consequently aggravating the inflammatory reaction and neuronal necrosis. In addition to rescuing and restoring neuronal cells, increasing evidence has shown that increasing the survival of endothelial cells, ameliorating brain angiogenesis, and mediating the recanalization of brain collaterals are great therapeutic targets. MSCs transplantation has been revealed to migrate to the peri-infarct region and differentiate into neuronal, glial, and endothelial cells to enhance neuroplasticity (30). Moreover, MSCs act in an indirect paracrine way as well. MSCs have also been shown to induce regenerative processes by increasing the level of insulin-like growth factor 1 (IGF-1) and inducing vascular endothelial growth factor (VEGF), angiopoietin-1 (Ang-1), essential fibroblast growth factor (bFGF), and neurotrophic factors in the host brain (48–51). These bioactive factors of VEGF and Ang-1 are the most essential in promoting neurological recovery by boosting neurogenesis. Besides that, the hypoxia and 0.04 MHz ultrasound-modified MSCs and MSCs-derived exosomes have been illustrated to have the capacity to achieve angiogenic effects (14, 52–54). Significantly, implantation of MSCs promoted angiogenesis and increased neurogenesis by releasing these angiogenic and neurotrophic factors. By conducting a three-dimensional analysis of the neovascularization in the peri-infarct region, Toyama et al. (55) and Chen et al. (56) demonstrated that the capillary-like tube formation was significantly induced in stroke mice treated with MSCs, suggesting a direct effect of MSCs on facilitating angiogenesis.

### MSCs support the post-stroke immunomodulatory effects

#### MSCs-microglia interactions

Microglia, which comprise a significant immune cell population in the central nervous system, appear as a ramified structure with a small soma in the resting form under physiological conditions (57, 58). When activated by ischemic stroke, microglia increase in number and transform to amoeboid forms characterized by the larger microglial cell body and shorter bumps. The activation of microglia activation is the first step in response to inflammation; further, the other immune cells, such as T cells, neutrophils, and natural killer cells, are activated (59, 60). While MSCs in microglial activation have been widely studied, there is not enough research on transplantation in ischemic stroke. Plenty of studies investigating various donor cell-derived MSCs identified a novel insight into crosstalk in ischemic stroke, and the role of MSCs in microglial activation has begun to be recognized (14, 61–63). For example, Yang et al. (64) indicated that bone marrow-MSCs can shift the microglia phenotype from M1 to M2, contributing to MSCs-induced brain repair. As a paracrine interaction between MSCs and microglia, the synergistic effect of MANF and PDGF-AA pathway governed M2 polarization. Furthermore, despite peripheral LPS treatment before the stroke, increased CD16/32-M1 microglia boosted the number of microglia surrounding the peri-infarct region and diminished CD206-M2 microglia on the post-stroke seventh day; they were rectified by the administration of human umbilical cord MSCs (65). Moreover, a series of researchers have accessed the effects of MSCs on microglial activation (14, 61–75); more details are shown in Table 1. To sum up, the application of MSCs appears to inhibit microglial activation and promote M2 polarization.

**Table 1:** Preclinical stroke studies assessing the effect of MSCs on the activation of microglia.

**Author**	**Year**	**Country**	**Species**	**Dosage**	**Route**	**MSCs source**	**The main effects on microglia**	**References**
Cunningham et al.	2020	UK.	Mice	1.4 x 10^6^	Sub	BM	Have no effect microglial Iba1 expression	(61)
Narantuya et al.	2010	Japan	Rats	NA	IV	BM	Reduce microglial activation and MMP level	(66)
Ishizaka et al.	2013	Japan	Rats	1 × 10^6^	IA	NA	Suppress microglia activation in the peri-infarct and core lesion	(67)
Yamaguchi et al.	2018	Japan	Rats	1 × 10^6^	IA	Blood	Suppress microglia activation in the peri-infarct cortex	(62)
Wang et al.	2014	China	Rats	2 × 10^6^	IV	BM	Inhibit macrophages/microglia activation in the ischemic brain	(68)
Wei et al.	2012	America	Rats	1 × 10^6^	IV	BM	Inhibit microglia activation in the ischemic brain	(14)
Nakajima et al.	2017	Japan	Rats	1 × 10^6^	IV	BM	Inhibit microglia activation and proinflammatory levels	(69)
McGuckin et al.	2013	France	Rats	NA	Stereotaxis	UC.	Decrease markers of microglial activation (lower ED1 and Iba)	(63)
Li et al.	2018	China	Rats	1 × 10^6^	IV	BM	Inhibit microglia activation	(70)
Lv et al.	2016	China	Cells	NA	NA	BM.	Inhibit hypoxia-activated rat microglia	(71)
Sheikh et al.	2019	Japan	Rats	3 × 10^6^	IV	BM	Inhibit microglia activation	(72)
Wang et al.	2013	Japan	Rats	3 × 10^6^	IV	BM	Inhibit microglia activation and proinflammatory gene levels	(73)
Yoo et al.	2013	South Korea	Rats	5 × 10^5^	Stereotaxis	BM.	Inhibit microglia activation	(74)
Sheikh et al.	2011	Japan	Rats	3 × 10^6^	IV	BM	Decrease the accumulation of Iba-1+ microglia	(75)
Feng et al.	2020	China	Mice	1 × 10^6/^20 g	IV	UC	Inhibit CD16/32-M1 microglia, Promote CD206-M2 microglia	(65)
Yang et al.	2020	China	Rats	1 × 10^6^	IV	BM	Induce M2 microglia polarization through PDGF-AA/MANF	(64)

NA, not available; IA, intraarterial; IV, intravenous; Sub, subcutaneous; BM, bone marrow; UC, umbilical cord; OGD, oxygen-glucose deprivation; MSCs, mesenchymal stem cell; IL, interleukin.

#### MSCs-neutrophils interactions

Neutrophils are the essential infiltrating cell type in the ischemic brain the first few days after stroke (76), tightly correlating with ischemic stroke-induced BBB disruption. The preclinical stroke studies have implied that MSCs' administration can reduce neutrophil accumulation in the brain. Vehicle or EVs (the equivalent of 2 × 106 MSCs) were intravenously administered to mice after transient intraluminal middle cerebral artery occlusion (77). MSC-EVs decreased specifically polymorphonuclear neutrophil infiltration in ischemic brains of aged mice. Moreover, MSCs can boost the beneficial effects of neutrophils on the brain. Bone marrow-MSCs can potentially induce interleukin-17 (IL-17) production in memory CD4^+^ T cells that, in turn, promote the enhanced phagocytic activity of neutrophils (78). Still, bone marrow-MSCs may also protect resting and interleukin-8-activated neutrophils from apoptosis, preserving their effector functions and suppressing the reactive oxygen species production (79).

#### MSCs-natural killer (NK) cells interactions

NK cells, one type of lymphocyte, belong to a part of the innate immune system that is well-known for the potential to mediate cytotoxicity and produce cytokines (80).

The immunomodulatory effects of MSCs on NK cells have been extensively studied in the peripheral regions. MSCs are involved in inhibiting the differentiation, proliferation, cytotoxicity, and activation of the NK cells through a variety of cytokines (81). These cytokines may include prostaglandin E2 (PGE2), soluble human leukocyte antigen-G5 (sHLA-G5), and transforming growth factor-β (TGF-β), which is partly linked to glycoprotein A repetitions predominant on the surface of MSCs (82). Additionally, hypoxic MSCs can also repress NK cell cytotoxicity and reduce the accumulation of host-derived NK cells when transplanted *in vivo*, as a result, contributing to ameliorating limb ischemia in allogeneic recipients (83).

#### MSCs-dendritic cells (DCs) interactions

The immune response to ischemic stroke consists of inflammatory and regulatory processes. DC is one of the cell types involved in innate and adaptive immunity. Upon an ischemic condition, the brain DCs are increased at 24- and 72-h post-stroke and accumulated in the peri-infarct region near invading T cells (84). Peripheral DC appearing in the brain was apparent at 72-h post-stroke and was confined primarily to the lesion core (84). MSCs are revealed to have capacities to suppress DCs differentiation and maturation and even reverse mature DCs to immature states (85–88). Gao et al. (85) indicated that MSCs inhibited the differentiation of human monocyte-derived DCs through both releasing IL-10 and direct cell contact. Likewise, Zhao et al. (87) showed that MSCs can differentiate mature DCs into a distinct regulatory DC population characterized by a lower expression of CD1a, CD80, CD86, and CD40 and a higher expression of CD11b. Importantly, such an effect on inhibiting DCs differentiation and maturity is demonstrated to be linked to both maintaining homeostasis of regulatory T cells and lower levels of proinflammatory cytokines TNF-α and MHC II surface antigens (86, 87).

#### MSCs-T cells interactions

T cells, which are involved in both innate and adaptive immune responses, can be divided into the αβ subset and the unconventional γδ subset (89). The αβ subset includes CD4^+^ T helper cells (Th1, Th2, Th17) that mainly modulate the functions of phagocytes and granulocytes, CD8^+^ T cells that have a cytotoxic role, and regulatory T cells (Treg) that regulate immune responses (89). After the ischemia-onset, T cells are revealed at the border of the infarct, where they appear within days (90, 91). More specifically, CD8^+^ T cells are recruited as early as 3 h post-ischemia onset, with CD4^+^ T cells and NK T cells following within 24 h, and accumulation of these T cells peaks 3 to 4 days after ictus (76, 92, 93). There is solid evidence that MSCs are linked to direct immunosuppressive properties via suppressing the activation and proliferation of CD4^+^ and CD8^+^ T cells while promoting activation, differentiation, and proliferation of Tregs through direct cell-to-cell communication or releasing of various factors. Upon a hypoxic-ischemic encephalopathy condition, MSCs can induce persistent peripheral T-cell tolerance and inhibit the invasion of T-cells into the preterm brain (94). During a critical limb ischemia condition, MSCs showed effective prevention of Th1 priming, which was strongly related to an altered IL-12/IL-10 production (95). Likewise, in renal ischemia/reperfusion rats, by releasing TGF-β, MSCs can not only suppress CD8^+^ T cells but boost the development of Tregs, as a result, repressing T cell-related inflammation (96). As such, MSCs might therefore contribute to suppressing the activation and proliferation of CD4^+^ and CD8^+^ T cells and promoting the proliferation of Tregs during an ischemic condition. However, information regarding this aspect of the interaction between MSCs and T cells upon an ischemic stroke condition appears to be limited. It is scarce, so additional and reliable data is urgently needed.

#### MSCs-B cells interactions

**Figure 1:**
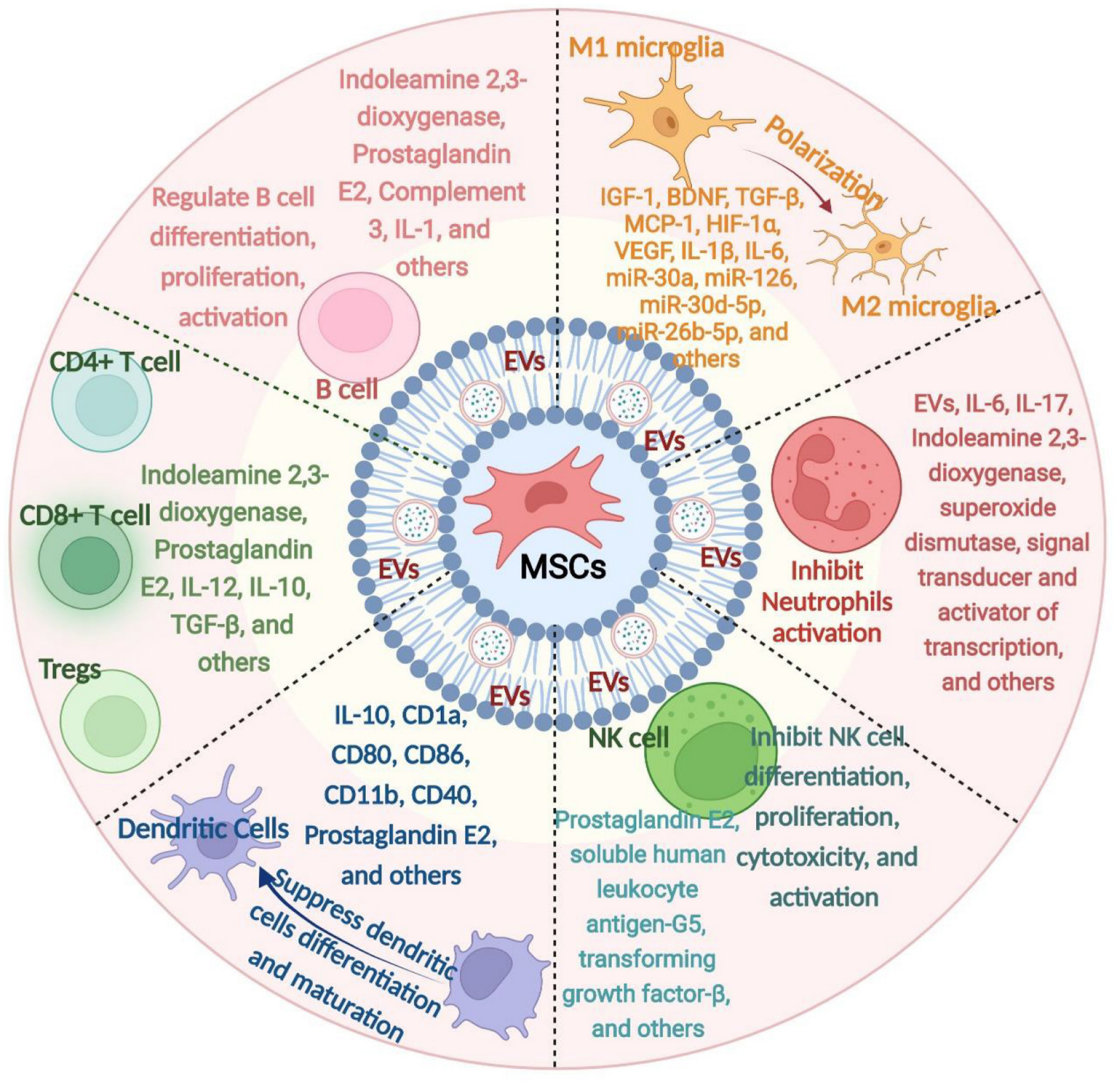
An overview of how mesenchymal stem cells (MSCs) interact with immune cells. MSCs derived from various tissue sources can release a series of mediators, which in turn interact with various immune cells, namely microglia, neutrophils, natural killer cells, dendritic cells, T cells, and B cells. IL, interleukin; EVs, extracellular vesicles; MSCs, mesenchymal stem cells; NK cell, Natural Killer cell; IGF-1, insulin-like growth factor 1; VEGF, vascular endothelial growth factor; bFGF, fibroblast growth factor; MCP-1, Monocyte chemoattractant protein-1; miR, microRNA; TGF-β, transforming growth factor-β.

B cells, one part of the adaptive immune response, have the capacity to present antigens, produce antibodies, and activate the immune system (97). These cells are detectable in insufficient quantities in the brain under normoxic conditions; however, they are trafficked in larger quantities to the brain tissues in response to injury (98, 99). B cell adoptive transfer to mice does not contribute to acute pathology but can support post-stroke recovery, independent of changing immune populations in recipient mice (100). Completed and ongoing clinical trials and preclinical studies on the therapeutic effects of MSCs transplantation against immune-mediated diseases have demonstrated an increased generation of B cells (101). The effectiveness of related MSCs-B cell interaction-based treatments dramatically depends on the functions of Bregs, as MSCs can increase the secretion of IL-10 by Bregs (101). On the contrary, several studies identified that MSCs are involved in suppressing the activation and proliferation of B cells. For instance, human adipose tissue-derived MSCs can inhibit the proliferation and chemotaxis of B cells by inducing cell cycle arrest in G0/G1 phase and regulating CXCR4 and CXCR5 expression, respectively (102). As such, *in vitro*, by secreting various factors, MSCs decreased the proliferation of B cells and the production of immunoglobulin (103). Taken together, the combined effects on the proliferation and activation of B cells are found in MSCs. However, the precise effect of inhibition/promotion on B cells modulated under ischemic stroke conditions is not fully clear yet. An overview of how MSCs interact with immune cells is shown in Figure 1.

### MSCs improve the post-stroke neurological function recovery

The size of the infarct volume is tightly correlated with ischemic stroke severity. *In vivo* experiments on MCAO rats demonstrated that MSCs derived from bone marrow, adipose tissue, and the umbilical cord could reduce the post-stroke infarct volume (104). However, many conditions, such as the source of MSCs, species injected, and the timing and dose of MSC injection, can affect specific effects on decreasing infarct volume after stroke. Along with such a reduction of infarct volume, the behavioral test analyses illustrated better test scores of mice/rats transplanted with MSCs at either time. Of note, this better test performance in the corner turn test, the rotarod test, balance beam test, tightrope test, and paw slips recording was long-lasting and stable until the end of the observation period (31, 105–107). It is suggested that MSCs can potentially mitigate postischemic motor coordination impairment in preclinical stroke experiments. Significantly, post-stroke impairment of the blood-brain barrier and perifocal vasogenic edema are also alleviated by endovascular MSCs administration. Post-stroke edema, impairment of the blood-brain barrier, as well as upregulation of aquaporin 4 (AQP4) water transport channels, play an essential role in the progression of ischemia and deteriorating disease recovery. Datta et al. (108) presented preliminary evidence that 1 × 10^5^ endovascular MSCs at 6 h post-stroke down-regulates AQP4 expression and alleviates vasogenic edema toward neuroprotection. Likewise, MSCs protected blood-brain barrier integrity by inhibiting the ischemia-induced astrocyte apoptosis, owing to the downregulation of AQP4 expression via the p38 signaling pathway (109).

## The underlying patterns of how MSCs exhibit the therapeutic effects

### MSC-EVs are critical players in treating ischemic stroke

EVs, the membrane-enclosed nanoscale particles secreted by all eukaryotes, always serve as a variety of molecular cargoes, such as peptides, lipids, proteins, and noncoding RNAs (43, 110). Based on the size of EVs, they can be divided into three subtypes: exosomes, microvesicles, and apoptotic bodies (111). Exosomes with a diameter of 30–150 nm form via the fusion of multivesicular bodies with membrane and are further released into the extracellular matrix (112, 113). Microvesicles with a diameter of 200–1,000 nm are produced owing to the outward budding of the plasma membrane (112, 113). Conversely, apoptotic bodies with a diameter of 1,000–5,000 nm are produced by dying cells and are even more abundant than the two other particles (111). Only exosomes and microvesicles are relevant to the therapeutic effects imparted by MSC-EVs. When these nanosized vesicles are released from donor cells into the extracellular matrix, they can be internalized by numerous recipient cells. In turn, they transfer the above bioactive cargos into recipient cells, including near and far from the secreting cell, further serving as messengers and performing biological functions. This cargo mix is revealed to mediate the biological properties of EVs and, indirectly, the treatment of MSCs under ischemic stroke conditions. The EVs derived from MSCs are emerging to be an appealing therapeutic tool for ischemic stroke, with the MSC-derived properties and the characteristics of effortless storage, lower immunogenicity, higher safety profile, and nature delivery vehicles. Previous research works indicated that EVs derived from MSCs promoted post-stroke recovery. They have the capacity to regulate the expression of recipient cell genes, alter cell properties involved in ischemic stroke, and mediate restorative effects, including cell survival, cell apoptosis, cell autophagy, angiogenesis, neurological function recovery, and immunomodulation, through a variety of molecular cargoes transfer (13, 31, 33, 34, 42, 53, 114–142). Moreover, by inhibiting the release of EVs, the beneficial effect on these aspects is also suppressed. For example, by establishing a coculture model that MSCs cocultured with hypoxic neurons and brain microvascular endothelial cells, the results showed that the MSCs treatment could inhibit the apoptosis of hypoxic neurons and restore the tube formation of brain microvascular endothelial cells (143). However, an inhibitor, GW4869, of EVs secretion can reverse these beneficial effects, indicating that these EVs are the key players that serve as the central mediator of the neuroprotective and angiogenic effects of MSCs (143). Currently, studies are paying attention to the function of the EVs isolated from bone marrow, adipose tissue, and, sometimes, umbilical cord-MSCs (144).

### The critical role of noncoding RNAs (NcRNAs) in treating ischemic stroke

Despite being well-established that most human RNA transcripts cannot encode proteins, the emerging evidence demonstrates that ncRNAs regulate cell physiology and shape cellular functions (145, 146). ncRNAs can be divided into long [namely long noncoding RNA (lncRNA) and circRNA] and small ncRNAs [including microRNAs (miRNAs), tRNAs, and piRNAs] by taking 200 nucleotides as the limit (147). miRNAs, ~18–24 nucleotides in size, are much earlier reported and the most discussed. lncRNAs are a large and heterogeneous kind of ncRNAs with more than 200 nucleotides and are involved in the modulation of transcription, translation, RNA metabolism, as well as homeostasis (148–150). CircRNAs are defined as circular covalently bonded structures associated with a higher tolerance to exonucleases (151), which serve as a scaffold for chromatin-modifying complexes, regulating the expression level of parental genes, modulating mRNA splicing, and acting as miRNA sponges (152, 153). Notably, the aberrant expression of many noncoding RNAs has been associated with aggressive pathologies. A variety of ncRNAs are reduced in the ischemic brain or blood after ischemic stroke, as previously reported for circSCMH1, miR-124-3p, miR-126, miR-221-3p, and miR-132 (114–119, 154), whereas other ncRNAs, namely miR-98 and miR-494, are increased at defined follow-up (155–157). MSC-based therapies offer an attractive approach because they promote cell survival, angiogenesis, and neurological function recovery, suppress cell apoptosis and autophagy, and regulate immunomodulation, where ncRNAs play an essential role. Interestingly, these ncRNAs were mainly derived from EVs, including lncRNA MALAT1, miR-1-3p, miR-17-92, miR-22-3p, miR-25, miR-26a, miR-26b-5p, miR-31, miR-124, miR-126, miR-132, miR-133b, miR-134, miR-138-5p, miR-146a-5p, miR-181b, miR-206, miR-210, miR-221-3p, miR-223-3p, miR-542-3p, and miR-1290 (31, 33, 34, 42, 114, 115, 117, 119, 121–125, 129–133, 135, 136, 138, 158). These EVs are isolated from bone marrow and adipose tissue, as well as umbilical cord-MSCs. Additionally, MSCs can regulate the expression of ncRNA directly, in turn, to support neuroprotection. For instance, Yang et al. indicated that MSCs-mediated mesencephalic astrocyte-derived neurotrophic factor paracrine signaling, the PDGF-AA/miR-30a^*^/XBP1/MANF pathway, synergistically mediates MSC-induced M2 polarization (64). Likewise, Huang et al. found that, with enhanced cell homing, MSCs can be applied to deliver miR-133b to boost the expression level of miR-133b in an ischemic lesion and further improve therapeutic effects (159). To sum up, MSCs can not only directly regulate the level of ncRNA but also indirectly regulate the level of ncRNA in the form of secreting exosomes, thus promoting the improvement of neurological function recovery. More details regarding preclinical studies that evaluate the effect of MSC-ncRNA on treating ischemic stroke are shown in Table 2 (31, 33, 34, 42, 64, 114, 115, 117, 119, 121–125, 129–133, 135, 136, 138, 158, 159).

**Table 2:** Preclinical studies evaluating the effect of MSC-non-coding RNA on treating ischemic stroke.

**Authors**	**Country, year**	**ncRNA**	**Expression**	**Source**	**Donor cell**	**Recipient cell**	**Main function**
Zhong and Luo (135)	China, 2021	miR-1-3p	Upregulation	EVs	ucMSCs	Primary neurons	Promote cell viability and inhibit apoptosis
El Bassit et al. (158)	USA, 2017	lncR MALAT1	Upregulation	EVs	MSCs	HT22 neuronal cells	Promote cell viability
Xin et al. (131)	China, 2017	miR-17-92	Upregulation	EVs	MSCs	Neurons, glial cells	Promote neuroplasticity
Zhang et al. (123)	China, 2021	miR-22-3p	Upregulation	EVs	ADSCs	Primary neurons	Promote cell viability and inhibit apoptosis
Kuang et al. (31)	Germany, 2020	miR-25	Upregulation	EVs	ADSCs	Primary neurons	Inhibit autophagy
Hou et al. (124)	China, 2021	miR-26a	Upregulation	EVs	ADSCs	Primary neurons	Promote cell viability and inhibit apoptosis
Ling et al. (132)	China, 2020	miR-26a	Upregulation	EVs	USCs	NSCs	Promote neurogenesis
Li et al. (122)	China, 2020	miR-26b-5p	Upregulation	EVs	ucMSCs	SH-SY5Y, PC12, microglia	Inhibit apoptosis and inflammation
Lv et al. (125)	China, 2020	miR-31	Upregulation	EVs	ADSCs	Primary neurons	Promote cell viability and inhibit apoptosis
Yang et al. (133)	China, 2017	miR-124	Upregulation	EVs	BMSCs	NPCs	Promote neurogenesis
Geng et al. (119)	China, 2019	miR-126	Upregulation	EVs	ADSCs	Neurons, ECs, BV2	Promote neurogenesis and inhibit inflammation
Feng et al. (114)	China, 2018	miR-132	Upregulation	EVs	BMSCs	Primary neurons	Promote cell viability and inhibit apoptosis
Xin et al. (121)	China, 2013	miR-133b	Upregulation	EVs	BMSCs	Neurons, AS	Promote neurite outgrowth
Xiao et al. (42)	China, 2018	miR-134	Downregulation	EVs	BMSCs	OLs	Inhibit apoptosis
Deng et al. (34)	China, 2019	miR-138-5p	Upregulation	EVs	BMSCs	Primary AS	Inhibit apoptosis
Zhang et al. (33)	China, 2021	miR-146a-5p	Upregulation	E.V.s	ucMSCs	BV2 microglia	Inhibit inflammation
Yang et al. (129)	China, 2018	miR-181b	Upregulation	EVs	ADSCs	BMECs	Promote angiogenesis
Zhong and Luo (135)	China, 2021	miR-206	Upregulation	EVs	ucMSCs	Primary neurons	Promote cell viability and inhibit apoptosis
Zhang et al. (130)	China, 2019	miR-210	Upregulation	EVs	BMSCs	BMECs	Promote angiogenesis
Ai et al. (115)	China, 2021	miR-221-3p	Upregulation	EVs	BMSCs	Primary neurons	Inhibit apoptosis and inflammation
Zhao et al. (136)	China, 2020	miR-223-3p	Upregulation	EVs	MSCs	BV2	Inhibit inflammation
Cai et al. (117)	China, 2021	miR-542-3p	Upregulation	EVs	MSCs	HA1800 AS	Inhibit apoptosis and inflammation
Yue et al. (138)	China, 2019	miR-1290	Upregulation	EVs	ucMSCs	Primary neurons	Inhibit apoptosis
Yang et al. (64)	China, 2020	miR-30a*	Upregulation	Cells	MSCs	Microglia	Inhibit inflammation
Huang et al. (159)	China, 2017	miR-133b	Upregulation	Cells	MSCs	Neurons/Astrocytes	Promote cell viability

BMSCs, Bone marrow-derived mesenchymal stem cells; ADSCs, adipose-derived stem cells; ucMSCs, umbilical cord mesenchymal stem cells; USCs, human urine-derived stem cells; EVs, Extracellular vesicles.

### The critical role of trophic factors and cytokines in treating ischemic stroke

**Figure 2:**
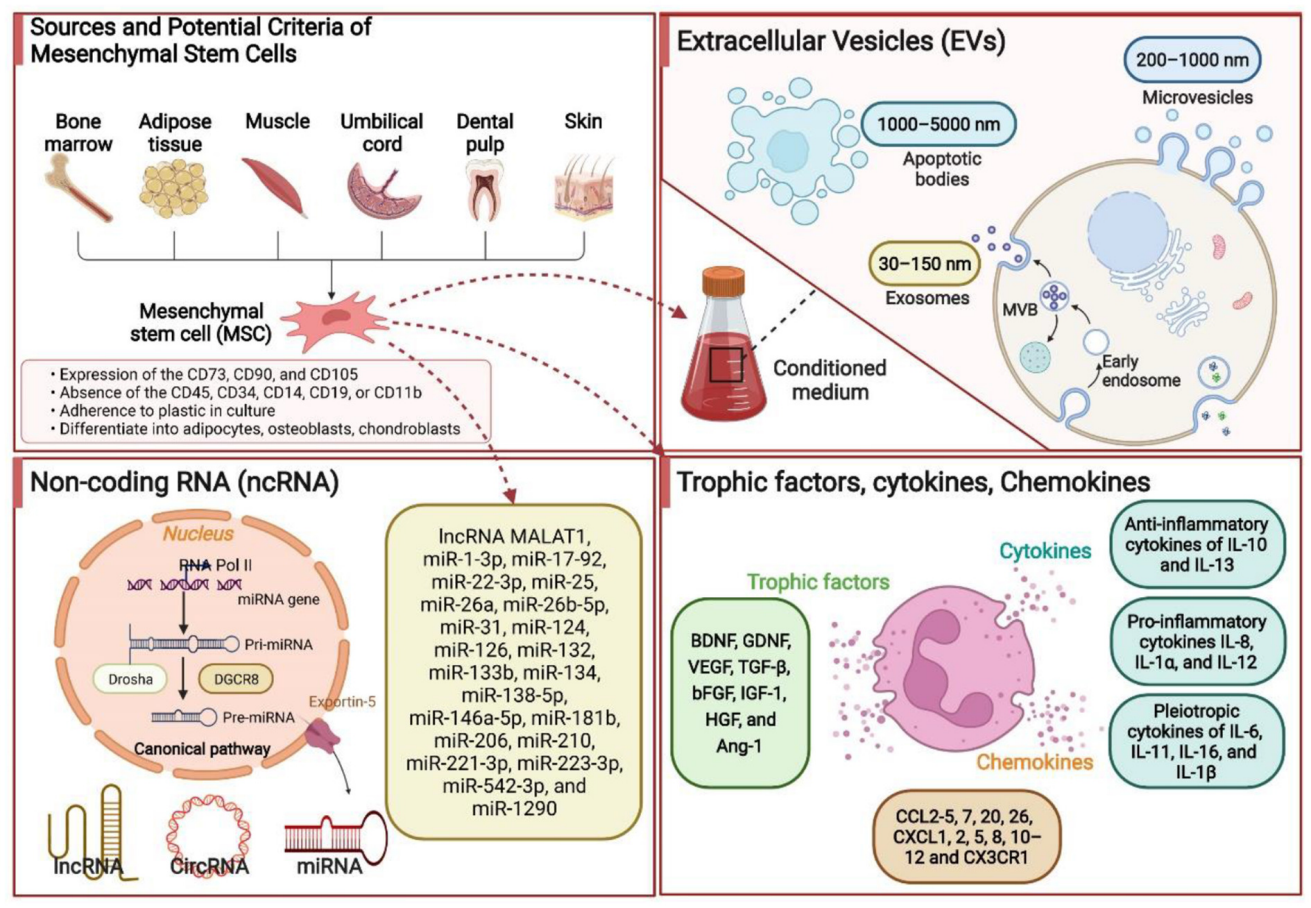
Some of the underlying therapeutic mechanisms associated with the mesenchymal stem cells (MSCs) in ischemic stroke. MSCs are isolated and identified from various tissue sources. These MSCs produce extracellular vesicles (EVs), noncoding RNAs (ncRNAs), trophic factors, chemokines, and cytokines by paracrine mechanisms to promote neurological recovery. Based on the EVs size, they can be divided into three subtypes, namely exosomes (30–150 nm), microvesicles (200–1,000 nm), and apoptotic bodies (1,000–5,000 nm). ncRNAs primarily include microRNA, long noncoding RNA, and circRNA. BDNF, brain-derived neurotrophic factor; GDNF, glial cell line-derived neurotrophic factor; VEGF, vascular endothelial growth factor; bFGF, basic fibroblast growth factor; IL, interleukin; TGF-β, transforming growth factor-β; HGF, hepatocyte growth factor; miR, microRNA.

Preclinical studies in rodent models of ischemic stroke have uncovered the potential effectiveness of the administration of trophic factors in ischemic brain injury recovery. The brain-derived neurotrophic factor (BDNF), glial cell line-derived neurotrophic factor (GDNF), and vascular endothelial growth factor (VEGF) are the most described (160). MSCs released or stimulated the release of three aforementioned neurotrophic factors associated with the contribution of ischemic stroke recovery. After administration, MSCs migrated from the vascular system outside the lesion to the area of the lesion core or peri-lesion to reduce the infarct volume by secreting BDNF, GDNF, and VEGF (161, 162). BDNF protein, highly expressed in the hippocampus, is known to affect the survival and proliferation of several neural cells, including cerebellar and cortical neurons (163). BDNF rapidly boosts in response to ischemic brain injury, contributing to reducing neuronal apoptosis and promoting neuronal survival (163). GDNF, produced by glial cells after brain injury, accelerates the survival and recovery of several types of mature neurons, including motor and dopaminergic neurons (164). VEGF, produced by neurons and astrocytes, is involved in various stages of neurodevelopment (proliferation, migration, differentiation, synaptogenesis, myelination (160). Additionally, VEGF stimulates angiogenesis by stimulating endothelial cell proliferation and migration and increases blood-brain barrier integrity (160). Notably, further growth and trophic factors, namely TGF-β, bFGF, IGF-1, HGF, and HGF, released or regulated by MSCs, are also involved in post-stroke neurological recovery. The types of cytokines released directly by MSCs or indirectly modulated in response to neuroinflammation due to stem cell transplantation are huge. They cannot be discussed in full detail here. Briefly, anti-inflammatory cytokines of IL-10 and IL-13, proinflammatory cytokines IL-8, IL-1α, and IL-12, and pleiotropic cytokines of IL-6, IL-11, IL-16, and IL-1β, correlated to immune function modulation after ischemic stroke, are revealed to be directly or indirectly produced by MSCs (165). In summary, MSCs played diverse therapeutic roles by secreting a series of trophic factors and cytokines. Hence, gene modification could be performed to enhance the therapeutic effects of MSCs by modulating the trophic factors and cytokines. However, attention should be given to the adverse effects of trophic factors and cytokines due to the adverse concentration. Some of the underlying therapeutic mechanisms associated with MSCs in ischemic stroke are summarized in Figure 2.

## Therapeutic application of stem cells in clinical ischemic stroke study

### Meta-analysis: The clinical application of MSCs in treating ischemic stroke

A comprehensive literature search of several electronic databases, namely PubMed, Cochrane Library, EMBASE, and Web of Science, was performed by two researchers independently from the inception of these databases to 30 June 2022. We retrieved studies assessing stem cells in treating ischemic stroke by adopting the following keywords: “stem cell” together with “ischemia,” “stroke,” “middle cerebral artery occlusion,” or “MCAO.” References from the identified reports were manually searched to identify other potential qualifying studies. The specific screening process is shown in Supplementary Figure 1. A total of 16 reports were included in this section from South Korea, India, the UK, China, the United States, Egypt, and Spain, which were conducted varied from 2005 to 2019 (166–181). Table 3, Supplementary Table 1 described the characteristics and quality assessment of included studies, respectively. The Stata, version 12.0, was used for endpoint analyses. When *I*^2^ > 50%, the data were deemed to have apparent heterogeneity, and a random-effect model was adopted. Otherwise, a fixed-effects model was adopted. Among all outcomes, weighted mean differences (WMD) or rate differences (RDs) with 95% CIs were applied for the assessment.

**Table 3:** Main characteristics of the clinical study assessing stem cells in treating ischemic stroke.

**References**	**Country**	**Study design**	**Sample size**	**Stem cell type**	**Cell dosage**	**Injection route**	**Follow-up**
Bhatia et al. (166)	India	RCT	20	Autologous BMMNCs	6.1 × 10^8^	IV	1 year
Bang et al. (181)	South Korea	RCT	30	Autologous MSCs	5 × 10^7^/2 times	IV	12 months
Meng et al. (179)	China	Non-RCT	120	Autologous MSCs	2.97 × 10^9^	IV	Half a year
Lee et al. (171)	South Korea	RCT	52	Autologous MSCs	5 × 10^7^/2 times	IV	5 years
Bhasin et al. (174)	India	Non-RCT	24	Autologous BMMNCs	5.46 × 10^7^	IV	24 weeks
Bhasin et al. (175)	India	Non-RCT	40	Autologous BMMNCs and MSCs	5.54 × 10^7^	IV	24 weeks
Prasad et al. (172)	India	RCT	120	Autologous MSCs	2.8 × 10^8^	IV	1 year
Chen et al. (167)	China	RCT	30	Autologous PBSCs	3–8 × 10^6^	IA.	Half a year
Bhasin et al. (176)	India	Non-RCT	20	Autologous BMMNCs	6.28 × 10^7^	IV	8 weeks
Ghali et al. (178)	Egypt	Non-RCT	39	Autologous BMMNCs	1 × 10^6^	IA	1 year
Bhasin et al. (177)	India	Non-RCT	12	Autologous MSCs	5–6 × 10^7^	IV	4 years
Hess et al. (169)	UK/USA	RCT	129	Allogeneic MAPC	1.2 × 10^9^	IV	1 year
Jin et al. (170)	China	RCT	20	Autologous BMMNCs	1 × 10^7^	Subarachnoid	7 years
Fang et al. (168)	China	RCT	16	Autologous EPSs and MSCs	2.5 × 10^6^/kg/2 times	IV	4 years
Savitz et al. (173)	USA	RCT	48	Autologous BM ALDHbr Cells	3.08 × 10^6^	IA	1 year
Moniche et al. (180)	Spain	Non-RCT	20	Autologous BMMNCs	3.38 × 10^6^	IA	Half a year

ALDHbr, aldehyde dehydrogenase; BMMNC, bone marrow-derived mononuclear cell; EPS, endothelial progenitor cell; IA, intra-arterial infusion; IV, intravenous infusion; MSCs, mesenchymal stem cells; PBSC, peripheral blood stem cell; MAPC, multipotent adult progenitor cells; RCT, randomized controlled trial.

**Figure 3:**
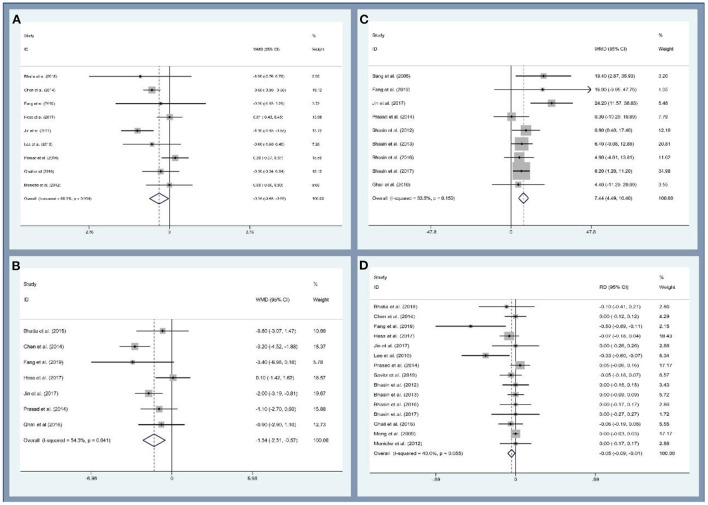
Forest plot for meta-analysis of the modified Rankin Scale (mRS) **(A)**, National Institute of Health Stroke Scale (NIHSC) **(B)**, Barthel index (BI) **(C)**, and death rate **(D)**.

First, this study analyzed the efficacy of MSCs on patients with ischemic stroke through the modified Rankin Scale (mRS), National Institutes of Health Stroke Scale (NIHSS), and Barthel index (BI). Data on mRS were provided by night studies. There are 219 and 227 participants in the MSCs and control groups. The patients treated with MSCs were associated with a statistically significant lower mRS value (WMD, −0.354; 95% CI, −0.681 to −0.027; *P* = 0.034, Figure 3A). Similarly, seven and nine studies reported the data of NIHSS and BI, respectively. The cross-sectional data from various studies were plotted and demonstrated that the NIHSS was statistically lower (WMD, −1.538; 95% CI, −2.506 to −0.571; *P* = 0.002, Figure 3B) and BI was statistically higher (WMD, 7.444; 95% CI, −4.488 to 10.401; *P* < 0.001, Figure 3C) in the MSCs group than that of the control group. Second, we also evaluated the safety of MSCs on patients with ischemic stroke; 15 studies (356 and 354 patients in the MSCs and control group, respectively) reported the death rate. No significant heterogeneity was observed, and a fix-effect model was used (*I*^2^ = 40%, *P* = 0.055). The death rate between the experimental and control groups was statistically significant (RD, −0.046; 95% CI, −0.086 to −0.005; *P* = 0.026, Figure 3D). More details regarding the results are described in Supplementary Table 2. Altogether, stem cell-based therapies have the capacity to improve neurological deficits and activities of daily living in patients with ischemic stroke. However, several common limitations exist for current studies, such as small sample size, long-term waiting for MSC culture, age of participants, heterogeneity of ischemic brain injury site, and severity (155, 156).

The clinical translation of MSC-based therapy for ischemic stroke is booming, and MSCs are expected to improve the sequence of ischemic stroke in patients. Nevertheless, this treatment has led to some controversy as well. (I) Stem cell translation has the potential to result in tumor formation (157). For example, stem cells derived from embryonic stem cells may have the potential for tumorigenicity. Moreover, a reduction of genetic modification of stem cells will be associated with a lower risk of tumor formation. (II) the controlled treatment of transplanted exogenous stem cells to regulate differentiation and achieve the desired therapeutic effect has yet to be studied (157, 182). (III) the insufficient brain delivery and retention and the invasiveness of current administration routes prevent MSCs from fully exerting their clinical therapeutic potential (183). (IV) the issue of immune rejection is also necessary to be addressed. Although MSCs rarely express the major histocompatibility complex, they can still cause some immunological issues (182).

## Conclusion

The application of MSCs in treating ischemic stroke is vast. In preclinical settings, the transplantation of MSCs offers an excellent opportunity for adjuvant ischemic stroke treatment, participating in multiple pathological processes, such as increasing cell survival angiogenesis and suppressing cell apoptosis and autophagy. Importantly, immunomodulation is another excellent target of MSCs by interacting with a variety of immune cells, namely microglia, neutrophils, NK cells, DCs, T cells, and B cells. However, no large-scale randomized, double-blind, multicenter clinical study exists to prove their effectiveness. In clinic, MSCs have many advantages: they are easy to harvest, expand, and store for a long time and are convenient to manage in many ways. Additionally, their clinical use does not raise many ethical issues. Increasing evidence supports the potential of MSCs to treat stroke, and autologous stem cell-based therapies can improve post-stroke neurological deficits and daily living activities in patients with minimal clinical adverse events. Nevertheless, the heterogeneity of MSCs is the primary barrier to their clinical application and therapeutic effect. Nonetheless, despite these issues, the application of MSCs appears to achieve neuroprotective effects, which result from the release of EVs and modification of various signaling pathways, such as ncRNAs, trophic factors, and cytokines.

## Author contributions

NT, MH, and YM designed and conceptualized the article. NT prepared the figures and tables. All authors significantly contributed to writing the paper and provided important intellectual content.

## Conflict of interest

The authors declare that the research was conducted in the absence of any commercial or financial relationships that could be construed as a potential conflict of interest.

## Publisher's note

All claims expressed in this article are solely those of the authors and do not necessarily represent those of their affiliated organizations, or those of the publisher, the editors and the reviewers. Any product that may be evaluated in this article, or claim that may be made by its manufacturer, is not guaranteed or endorsed by the publisher.
