# Supplementary material for: Mesenchymal stem cell therapy for ischemic stroke: Novel insight into the crosstalk with immune cells
Source: Front Neurol. 2022 Nov 8;13:1048113. doi: 10.3389/fneur.2022.1048113 (PMC9679024; doi:10.3389/fneur.2022.1048113)
Supplement: Supplementary file 1 [file Data_Sheet_1.docx]

**Supplements of Figures**


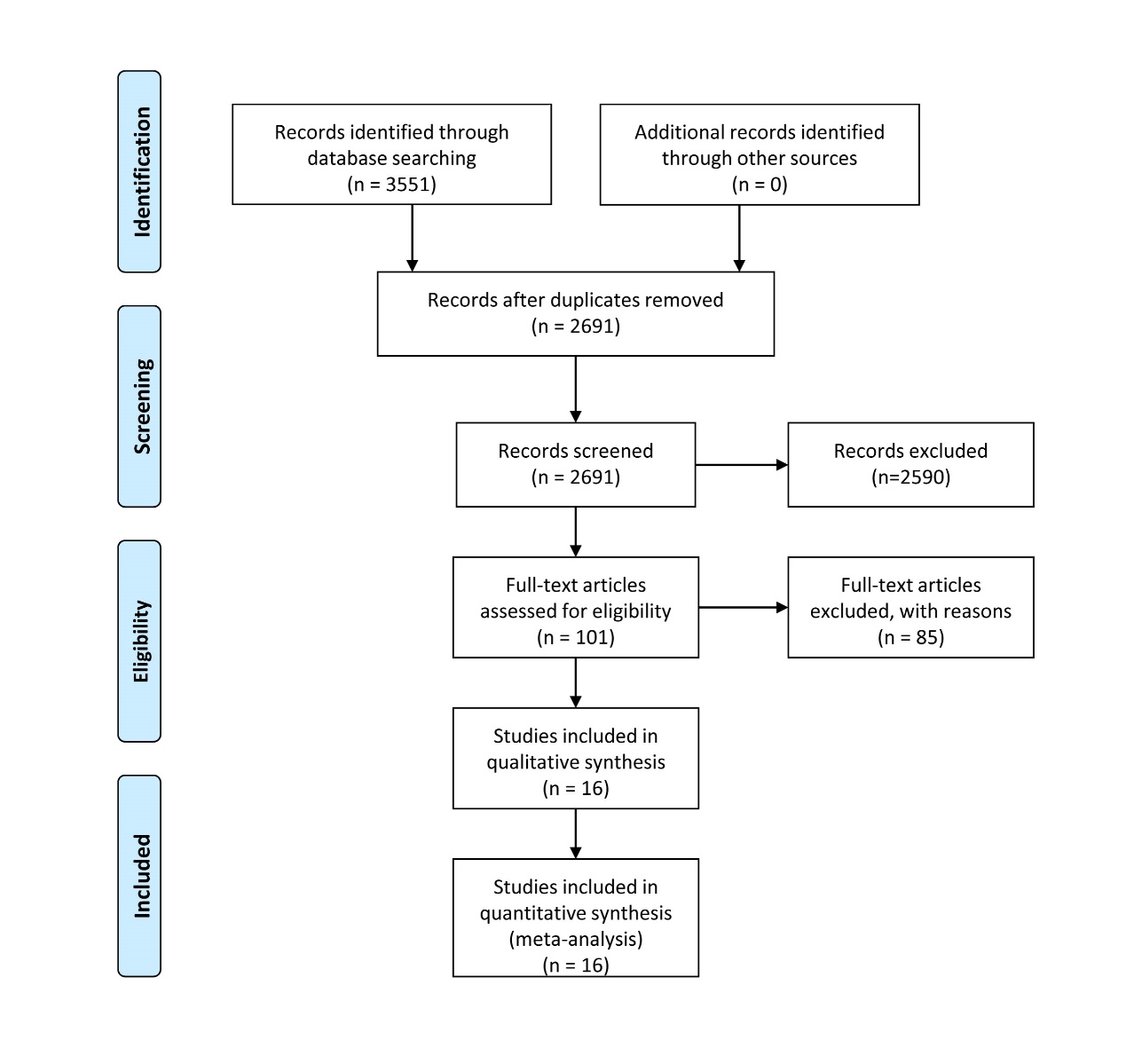


**eFigure1:** **Flowchart of the clinical trials selection process.**

**Supplements of Tables**

**eTable1.** **The quality assessment of included trials.**

| **Author, year** | **Design** | **Newcastle-Ottawa Scale (NOS)** | | | |
| --- | --- | --- | --- | --- | --- |
|  |  | **Selection** | **Comparability** | **Exposure** | **Total Score** |
| **Bhatia et al.** | 2018 | 3 | 1 | 3 | 7 |
| **Chen et al.** | 2014 | 3 | 2 | 1 | 6 |
| **Fang et al.** | 2019 | 4 | 1 | 2 | 7 |
| **Hess et al.** | 2017 | 3 | 2 | 2 | 7 |
| **Jin et al.** | 2017 | 4 | 1 | 2 | 7 |
| **Lee et al.** | 2010 | 2 | 2 | 3 | 7 |
| **Prasad et al.** | 2014 | 4 | 1 | 2 | 7 |
| **Savitz et al.** | 2019 | 4 | 2 | 1 | 7 |
| **Bhasin et al.** | 2012 | 3 | 1 | 2 | 6 |
| **Bhasin et al.** | 2013 | 3 | 2 | 2 | 7 |
| **Bhasin et al.** | 2016 | 3 | 1 | 3 | 7 |
| **Bhasin et al.** | 2017 | 2 | 2 | 3 | 7 |
| **Ghali et al.** | 2016 | 2 | 3 | 2 | 7 |
| **Meng et al.** | 2009 | 3 | 2 | 3 | 8 |
| **Moniche et al.** | 2012 | 3 | 2 | 3 | 8 |
| **Bang et al.** | 2005 | 2 | 1 | 2 | 5 |

**Note: NOS= Newcastle-Ottawa scale.**

**eTable2. The outcomes of this meta-analysis.**

| **Outcomes** | **Studies**  **Numbers** | **Sample size** | | **Overall effect** | | | **Heterogeneity** | |
| --- | --- | --- | --- | --- | --- | --- | --- | --- |
|  |  | **Experiment** | **Control** | **Effect estimates** | **95% CIs** | **p-Value** | **I^2^(%)** | **p-Value** |
| **Therapeutic Application of Stem Cells in Clinical Ischemic Stroke Study** | | | | | | | | |
| **Modified Rankin Scale** | 9 | 219 | 227 | WMD=-0.354 | -0.681 to -0.027 | P =0.034 | 58.1% | P=0.014 |
| **National Institute of Health stroke scale** | 7 | 193 | 181 | WMD=-1.538 | -2.506 to -0.571 | P =0.002 | 54.3% | P=0.041 |
| **Barthel index** | 9 | 159 | 162 | WMD=7.444 | 4.488 to 10.401 | P<0.001 | 33.5% | P=0.150 |
| **Death incidence** | 15 | 356 | 354 | RD=-0.046 | -0.086 to -0.005 | P=0.026 | 40% | P=0.055 |

Note: CIs = confidence intervals; RD = rate difference; WMD= weighted mean differences.
